# Burnout syndrome in medical oncologists during the COVID-19 pandemic: Argentinian national survey

**DOI:** 10.3332/ecancer.2021.1213

**Published:** 2021-03-25

**Authors:** Andres Guercovich, Gonzalo Piazzioni, Federico Waisberg, Pablo Mandó, Martín Angel

**Affiliations:** 1 Centro Oncológico Integral (COI), Neuquén Q8300XAC, Argentina; 2 Psychologist, Instituto Austral de Salud Mental, Neuquén Q8300XAC, Argentina; 3 Instituto Alexander Fleming, CABA, Buenos Aires C1426ANZ, Argentina; 4 Breast and Gynecological Tumor Unit, Centro Universitario CEMIC, CABA, Buenos Aires C1431FWN, Argentina; 5 Clinical Oncologist, GenitoUrinary Tumor Unit, Instituto Alexander Fleming, CABA, Buenos Aires C1426ANZ, Argentina; * Member of Asociación Argentina de Oncología Clínica (AAOC); a https://orcid.org/0000-0002-1463-8887

**Keywords:** burnout, pandemic, COVID-19

## Abstract

Burnout (BO) syndrome is a condition that results in physical and mental distress. The current COVID-19 pandemic is strongly affecting the mental health of the general population. We aimed to assess the incidence of BO among medical oncologists and determine factors associated with burnout levels during the current pandemic.

**Methods:**

A digital survey was created for this study. The Spanish-validated version of Maslach BO Inventory was incorporated to define BO. Social and demographic information was analysed to remove duplicated answers.

**Results:**

A total of 188 Argentinian medical oncologists from 16 cities participated in the survey. The median age of the participants was 43 years (IQR 38-50) and a similar distribution between male and female was observed. At the time of the survey, Argentina was in the third month of strict lockdown. Most of the participants practiced in both public and private practice facilities (55.3%) and the majority reported more than 10 years of experience (53.2%). Twenty-five percent (43) of subjects reported high levels of DP, 39.9% (75) reported high levels of EE and 53.7% (101) reported low levels of PA. BO Maslach criteria were fulfilled by 14.9% (28). We compared this result with other burnout assessment tools. Using the Gil-Monte and Neira tool, BO-associated domains were altered in 77.1%, 42% and 42% for EE, DP, and PA domains, respectively. Concomitantly, under Neira assessment a domain impairment was appreciated in 77.1%, 76% and 54% respectively. BO criteria were met by 30.3% (57) according to Gil-Monte and 47.9% (90) to Neira.

**Conclusion:**

BO is a multifaceted issue with a negative impact on physicians, patients, and institutions. During the COVID-19 pandemic, BO criteria was met in a considerable proportion of survey respondents using MBI, and Peiro and Neiro tools and younger age, use of antidepressants and psychological medications and income reduction arose as statistically significant factors after multivariate analysis.

## Introduction

During the 1970s, the psychologist Herbert Freudenberger described a condition that occurs when work coupled with additional life pressures exceeds the ability to cope, resulting in physical and mental distress and since then was named burnout (BO) syndrome [1].

Oncologists are particularly at increased risk for mental health distress due to the emotionally demanding nature of their work, grieving much more frequently than other specialities [2].

The current COVID-19 health crisis is strongly affecting the mental health of the general population. In particular, the pandemic has been associated with psychological distress and collateral concerns for parents in lockdown, due to unstable financial circumstances, school closures and suspended educational services for children. The lack of formal recommendations regarding how oncological patients should be treated has added a substantial factor to increase BO risk in oncologists [3, 4]. 

Considering classic characterisations, BO typically implicates physical and mental exhaustion, depersonalisation (DP) and cynicism. It may also include specific clinical alterations, such as insomnia, cognitive impairment and cardiovascular and gastrointestinal related symptoms. Furthermore, specific subgroups are at increased risk of BO syndrome, including female patients, age lower than 40, younger or non-partnered physicians and providers with long working hours. Cancer healthcare providers have been largely associated with increased risk of BO in comparison to other medical specialisations.

In this unique worldwide context, BO should be routinely addressed and recognised as an expectable turn-out of coping with patients during the COVID-19 pandemic. Early recognition of associated symptoms may prevent stress and health related alterations in healthcare providers, and in turn, improve patient’s experience during cancer diagnosis and treatment. While different strategies may be applicable to address BO, such as discussing resilience methods, a natural first step is enhancing self-awareness and providing safe structures to discuss this occupational health issue.

Under these special circumstances, we aimed to assess the incidence of BO among medical oncologists and determine sociodemographic and occupational factors associated with elevated BO levels during current pandemic.

## Methods

### Subjects

A digital survey was created for the purposes of this study. Subjects that were members of the Argentinean Society of Clinical Oncology were invited to answer the survey by email. Two emails were sent to request collaboration, contemplating the database of existing members up to 30 June 2020. Included subjects were cancer-specialised physicians or fellows that had completed all the requested items in the survey. Duplicated answers were removed. The Argentinean Association of Clinical Oncology approved the use of retrieved information for investigational purposes.

### Survey design and data collection

The study survey was designed in Google forms and included social, demographic and work-related information, including practice setting and years of experience. The Spanish-validated version of Maslach BO Inventory (MBI) [5], which is designed to measure the three stages of emotional exhaustion (EE), DP and personal accomplishment (PA) was incorporated to define BO. The EE subscale describes feelings of being emotionally exhausted because of the work and contains nine items. The PA subscale contains eight items that describe beliefs of competence and successful achievement at work. The DP subscale describes detached and impersonal treatment of patients and consists of five items. Each of the 22 items asks respondents to describe their feelings on a 7-point scale. The survey was conducted between 20 May and 30 June 2020. Evaluated domains were considered disturbed, according to MBI, if the sum of corresponding questions was ≥27 or 13, or ≤31 in EE, DP or PA stages, respectively. A respondent was considered to meet BO definition if the three domains were altered. We also evaluated MBI and BO definition according to other popular scales, including Gil-Monte and Peiro [6] and Neira [7] assessments, with cutoffs of 25/9/35 and 22/5/35, respectively.

All the data in this survey were collected anonymously, and no personal information was requested in the survey. Social and demographic information was analysed to remove duplicated answers. The full survey can be accessed at https://bit.ly/32HRwUH.

### Statistical analysis

Categorical variables were expressed as absolute numbers and percentages. Continuous variables were described in terms of means and standard deviations if normally distributed or medians and interquartile ranges (IQR) otherwise. Comparisons among groups were conducted using the Student *t* test and Wilcoxon rank sum test depending on distribution for continuous variables, and χ2 test and Fisher exact test for categorical variables. Logistic regression analysis was performed to evaluate the independent association between BO syndrome and demographic and practice setting characteristics. A *p* value < 0.05 was considered statistically significant. Statistical analysis was performed with STATA 14 (STATA, College Station, TX). Descriptive data and results were graphed using GraphPad v7.00 (GraphPad Software, USA).

## Results

### Participant demographics

A total of 188 Argentinian medical oncologists from 16 cities participated in the survey. The median age of the participants was 43 years (IQR: 38–50) and a similar distribution between male and female was observed (50.5% versus 49.5%). At the time of the survey, Argentina was in the third month of strict lockdown. Most of the participants practised in both public and private practice facilities (55.3%) and the majority reported more than 10 years of experience (53.2%), being only 8.5% in-training fellows. Demographics and occupational characteristics are further described in Table 1.

### Psychological well-being during COVID-19 pandemic

Several aspects of psychological well-being were asked in the survey (Table 2). Respondents presented 17.0% use of antidepressants or sleeping drugs. The financial implications of the COVID-19 pandemic were a specific issue highlighted by most survey respondents. Alarmingly, 72.3% of included physicians have answered that family income was reduced during COVID-19 pandemic, and in 36% of total professionals, the decrease was higher than 20%. 52% of cancer healthcare providers reported working hours that exceeded the average of 8 hours per day and almost half worked on weekends. Concomitantly, a lack of financial or psychological support by their primary institutions was appreciated by 58.0% and 71.3% of survey respondents, respectively. Nevertheless, 90.4% of oncologists with BO would choose this speciality again if they had the opportunity. Regarding to COVID-19 patients, 28.7% reported that they did not have support with personal protection equipment but 81.9% felt prepared to treat patients with COVID-19.

### MBI results

**Figure 1.**
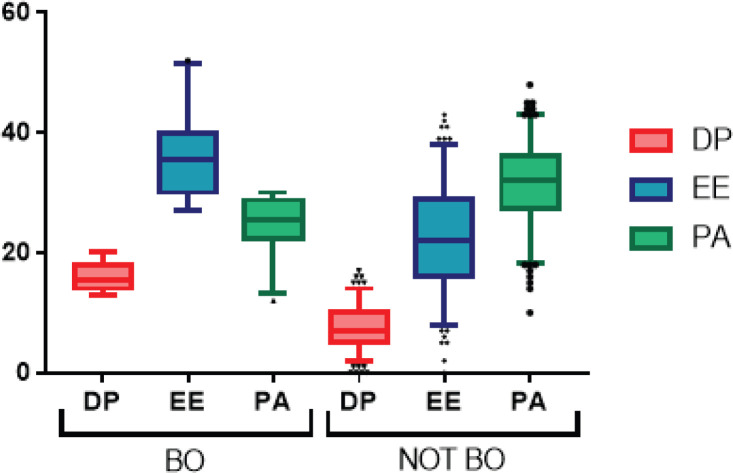
Maslach Burnout Inventory (MSI) results according to BO.

**Figure 2.**
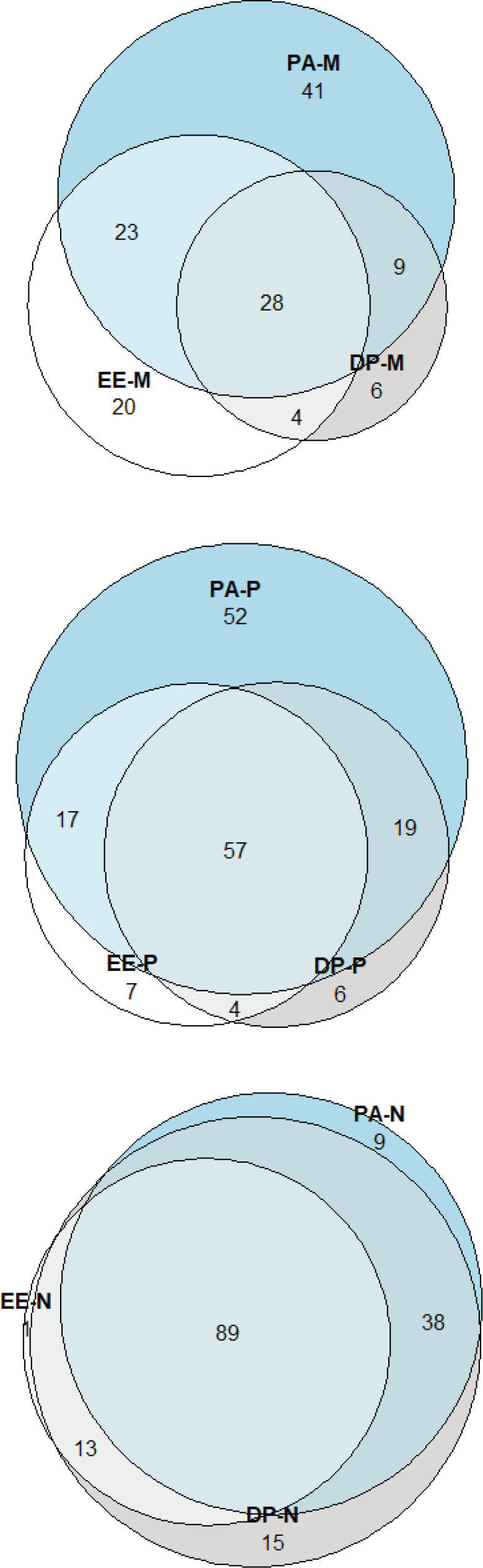
Altered domains according to assessment tool. (a): MSI, (b): Gil-Monte and Peiro and (c): Neira.

MBI results are presented in Table 3. 25% (43) of subjects reported high levels of DP, 39.9% (75) high levels of EE and 53.7% (101) low levels of PA. BO Maslach criteria were fulfilled by 14.9% (28). Domain results in subjects according to BO are presented in Figure 1. Among subjects considered not to present BO criteria, high levels of DP (11.9%) and EE (29.4%) were detected, as well as low levels of PA (45.6%). We compared this result with other BO assessment tools. Using the Gil-Monte and Neira tool, BO-associated domains were altered in 77.1%, 42% and 42% for EE, DP and PA domains, respectively. Concomitantly, under Neira assessment, a domain impairment was appreciated in 77.1%, 76% and 54%, respectively. BO criteria were met by 30.3% (57) according to Gil-Monte and 47.9% (90) to Neira. Altered domains according to assessment tool are shown in Figure 2.

### Factors associated with BO

Univariate and multivariate analysis were performed to identify prognostic factors for BO in our population. In the univariate analysis, it was determined that age over 40 years old, having children and psycho-oncological assistance could be considered protective factors and weekend working hours, use of antidepressants and reduced income during the COVID-19 pandemic were risk factors for BO. Importantly, age over 40 years old (OR: 0.31, 95% CI: 0.13–0.75, *p* = 0.01), use of antidepressants and sleeping pills (OR: 3.33, 95% CI: 1.21–9.16, *p* = 0.02) and reduced income during COVID-19 pandemic (OR: 3.44, 95% CI: 1.08–10.99, *p* = 0.04) remained statistically significant after multivariate analysis (Tables 4 and 5).

## Discussion

BO is prevalent among cancer health providers. As presented by Telzaff *et al* [8], BO has increased among oncologists from 2015 to 2019. In fact, in 2019, 48.7% of 234 American Society of Clinical Oncology (ASCO) attendees reported BO with alterations in at least one domain of the MBI Subscale. 17.1% of the total population had both high levels of EE and DP. Specifically, authors highlighted that BO was associated with the increase in working hours, economical compensation, relationship with other professionals and sub-specialisation.

In this context, it is essential to remind that providing adequate healthcare includes supporting physicians during challenging times. During the COVID-19 pandemic, many physicians may have faced unexpectedly dramatical situations, such as coping with hospitalisation and death of coworkers and cohabitants, dealing with patients suffering in isolation, fearing of getting exposed to COVID-19 and adapting to new work scenarios.

A recent survey conducted by the European Society for Medical Oncology (ESMO) [9] included 1,520 respondents from 101 countries, and reported that 38% of participants had experienced feelings related with BO, and 78% expressed concern for personal safety. In a follow-up survey, done between July and August 2020, the incidence of BO increased from 35% to 49%.

Compared with other health care professionals, the incidence of BO during the COVID-19 pandemic was not different from that observed, as reported in the Lasalvia *et al* [10] study, where more than 1,900 health professionals were evaluated. Likewise, the same risk factors were identified as in the oncologist population evaluated by our group. Several changes in daily practice were documented by the ESMO Resilience Task Force including the increase of remote consultations (50%), COVID-19 inpatient attention (14%) and reduction in research activity (38%). Factors associated with BO included currently receiving psychological care, increased number of hours or out of hours work and concern about impact on career or personal wellbeing.

Furthermore, during the COVID-19 pandemic, Hilmi *et al* [11] recently reported that 32% and 17% of 222 medical and radiation oncology residents could be classified as anxious or depressed, respectively, in accordance to Hospital Anxiety and Depression Score. Alarmingly, an increase of tobacco, alcohol and psychostimulant drugs was referred by 31%, 24% and 29% of respective current users and 31% of the respondents answered that they had not received adequate protective equipment.

Noticeably, BO has a meaningful impact among Argentinean healthcare providers. A very interesting study conducted by Suñer-Soler *et al* [12], which included 7,503 Argentineans of a total 11,530 Spanish-speaking professionals between 2006 and 2007, revealed that Argentina was one of the countries with lower scores of job satisfaction, optimism and personal economy. In that subgroup, nearly 80% of the respondents expressed that working conditions may have affected family wellbeing, 87% associated working conditions with treatment errors and 64% considered changing their profession. Our results highlighted that a comparable proportion of physicians could be experiencing BO according to at least one of the domains of MBI. DP and PA were the most affected domains. PA may result in an uphill struggle for physicians during the pandemic. Particularly, in the MBI, subjects are asked whether they were energetic, able to create a relaxed atmosphere or whether they were positively influencing others. DP may arise as an unwanted mechanism to cope with stressful situations, and may be associated with a more direct impact on the quality of patient care.

Despite the lack of adequate sources, it needs to be considered that oncologists in Latin America face worse working conditions than cancer specialists of High Income Countries [13]. The lockdown has brought critical consequences in Low- and Middle-Income Countries economies, and particularly in Argentina, restrictive measures lasted for almost 8 months. In addition to the reduction of patients’ routine visits, many physicians may have experienced an unexpected increase of telephone consultations, which in many cases, were not economically compensated. Under these circumstances, our study results reflect a contrasting scenario in our population when compared with available publications during the COVID-19 pandemic. The reduction of income represented one of the principal factors for BO after the multivariate analysis. Lowered incomes may also be a cause of the particularly high prevalence among younger cancer professionals in our sample.

Social support, psychological guidance, group discussions and experience sharing with other professionals, stress management techniques and the maintenance of a healthy lifestyle, including routinely exercising, protected times for leisure activities and resting are general recommendations to cope with BO [14]. Notably, individual and group strategies such as mindfulness or participation in Balint groups have been associated with lower incidence of BO alike [15, 16]. While resilience skills can be worked, this does not reduce the responsibility of employers to promote measures to improve health professional’s wellbeing. BO may prove to be a group rather than an individual experience and it is necessary to adequately understand causes that motivated distress among healthcare providers. In addition to maintaining fair incomes, gender equality and protected times for other activities apart from patient care, it becomes essential to incorporate BO into work culture, raise self-awareness and avoid punishing a natural outcome in a challenging time.In this challenging setting, professional associations play a key role to defend labour rights in situations where physicians are at risk of being more characterised as altruistic or disinterested providers rather than jobholders that deserve fair compensation. This should be emphasised, considering that the majority of respondents expressed defective psychological and financial support by their primary institutions.

Fortunately, only 9.6% of cancer professionals in our survey expressed that given the chance they would not choose oncology again. This encouraging aspect can be influenced by a high proportion of respondents with less than 10 years of medical practice. Nevertheless, efforts to preserve cancer professionals and promote work-life balance need to be addressed by National Cancer Programmes, especially considering that cancer incidence is expected to increase 66.5% in South America in 2030 [17]. Pandemic crises may have contributed to bring to light the stressing reality that many cancer care providers commonly experience. Undeniably, more efforts should be pursued to preserve the wellbeing of cancer professionals and BO management needs to be prioritised in the global health agenda.

Our results should be carefully analysed given the study limitations. Our sample was mainly conformed by relatively young physicians that worked in Private or Private and Public facilities. Also, most respondents work and live in the most populated cities of our country, which hampers study representativeness. Compassion fatigue (CF) was not evaluated in our study, but it would be interesting to be able to evaluate it in future studies that include CF rating scales in oncologists and other health workers.

## Conclusions

BO is a multifaceted issue with a negative impact on physicians, patients and institutions. During the COVID-19 pandemic, the criteria for BO were met in a considerable proportion of survey respondents using MBI, and Peiro and Neiro tools and younger age, use of antidepressants and psychological medications and income reduction arose as statistically significant factors after multivariate analysis. Given the high prevalence of physician distress and its potential repercussions for quality of care and the rising cancer incidence in South America, healthcare providers, professional associations, health researchers and policy makers should incorporate BO awareness and management as a strategic area of National Cancer Programmes.

## Conflicts of interest

None of the authors have conflicts of interest to declare.

## Funding

The authors have not declared a specific grant for this research from any funding agency in the public, commercial or not-for-profit sectors.

## Figures and Tables

**Table 1.** Demographics characteristics (*n*:188).

Age, median (IQR)	43 (38–50)
Sex, *n* (%)	Male 93 (49.5)
	Female 95 (50.5)
Marital status, *n* (%)	Single 39 (20.7)
	Couple not married 43 (22.9)
	Married, 106 (56.4)
Children, *n* (%)	137 (72.9)
Children at school, *n* (%)	72 (38.3)
Daily physical activity, *n* (%)	122 (64.9)
Occupational characteristics	
Primary place of work, *n* (%)	Private, 60 (31.9)
	Public, 24 (12.8)
	Both, 104 (55.3)
Years of practice, *n* (%)	Resident, 16 (8.5)
	<5 years 23 (12.2)
	5–10 years 49 (26.1)
	>10 years, 100 (53.2)
Working hours per week, *n* (%)	≤25 hours, 3 (1.6)
	26–40, 86 (45.7)
	>40, 99 (52.7)
Working on weekends, *n* (%)	82 (43.6)
Number of patients per week *n* (%)	Under 50, 38 (20.2)
	51–100, 130 (69.2)
	More 100, 20 (10.6)
Clinical research *n* (%)	86 (45.7)
Telemedicine, *n* (%)	Less 20%, 120 (63.8)
	21%–50%, 53 (28.2)
	51%–80%, 13 (6.9)
	More 80%, 2 (1.1)
Designated hour/week to read about COVID-19, *n* (%)	1–5, 129 (68.6)
	6–10, 50 (26.6)
	>10, 9 (4.8)

**Table 2.** Psychological wellbeing.

Use of antidepressant or sleeping drugs	32 (17.0)
Family income reduced by COVID-19 pandemic	136 (72.3)
Medical institution support	
Financial	79 (42.0)
Personal protective equipment (PPE)	134 (71.3)
Psychological	54 (28.7)
AAOC support	
Academic	125 (66.5)
COVID-19 pandemic	148 (78.7)
Feeling of being prepared to treat patients with COVID 19	154 (81.9)

**Table 3.** MBI results.

DP, median (IQR)	8 (6–12.5)
High (≥13)	47 (25.0)
Intermediate (7–12)	78 (41.5)
Low (≤6)	63 (33.5)
EE, median (IQR)	23 (18–31)
High (≥27)	75 (39.9)
Intermediate (17–26)	70 (37.2)
Low (≤16)	43 (22.9)
PA, median (IQR)	31 (26–35)
High (≥39)	101 (53.7)
Intermediate (32–38)	62 (33.0)
Low (≤31)	101 (53.7)
BO Maslach	28 (14.9)
BO Peiro	57 (30.3)
BO Neira	90 (47.9)

**Table 4.** Univariate analysis of factors associated with BO syndrome.

Variables	OR	95% CI	*p*-value
Age > 40 years old	0.43	0.19–0.96	0.040
Parenthood	0.43	0.19–0.99	0.046
Working on weekends	2.70	1.17–6.22	0.020
Psycho-oncology	0.48	0.21–1.09	0.078
Antidepressants and sleeping pills	2.27	0.90–5.73	0.084
Reduced income	2.57	0.85–7.81	0.096

**Table 5.** Multivariate analysis of factors associated with BO syndrome.

Variables	OR	95% CI	*p*-value
Age > 40 years old	0.31	0.13–0.75	0.009
Antidepressants and sleeping pills	3.33	1.21–9.16	0.019
Reduced income	3.44	1.08–10.99	0.037
